# Supplementary material for: The interactome of intact mitochondria by cross-linking mass spectrometry provides evidence for coexisting respiratory supercomplexes
Source: Mol Cell Proteomics. 2017 Dec 8;17(2):216–32. doi: 10.1074/mcp.RA117.000470 (PMC5795388; doi:10.1074/mcp.RA117.000470)
Supplement: Supplemental Data [file supp_RA117.000470_133922_0_supp_23978_fzffwf.zip › spectra_annotation/mito_spectra_annotation/168-1-2-1-3-1.pdf]

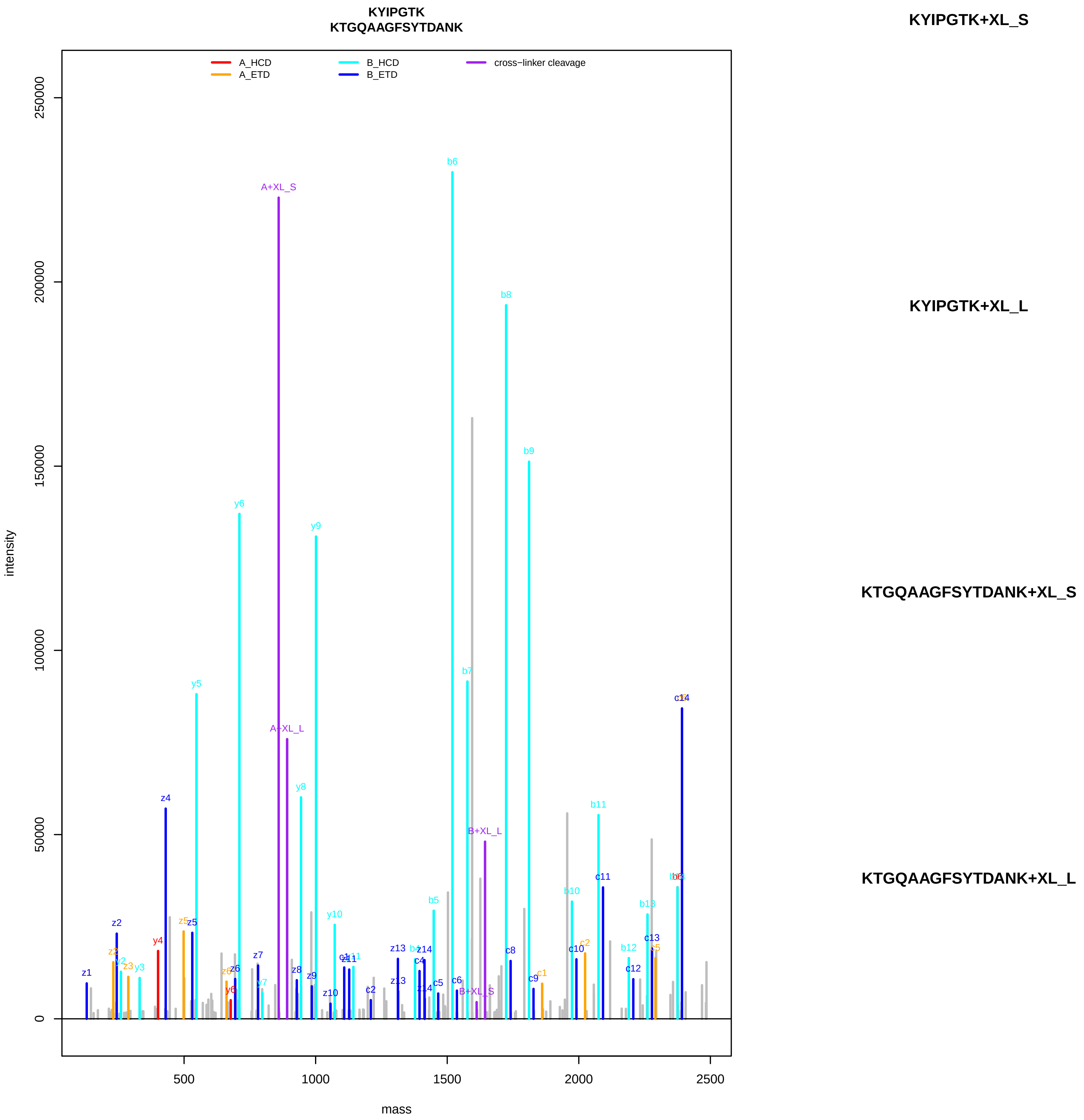
KYIPGTK
KTGQAAGFSYTDANK
A_HCD
A_ETD
B_HCD
B_ETD
cross−linker cleavage
intensity
mass
0
50000
100000
150000
200000
250000
500
1000
1500
2000
2500
A+XL_S
A+XL_L
B+XL_L
B+XL_S
b5
b6
b7
b8
b9
b10
b11
b12
b13
y2
y3
y4
y5
y6
y7
y8
y9
y10
z1
z2
z3
z4
z5
z6
z7
z8
z9
z10
z11
z13
z14
c1
c2
c4
c5
c6
c8
c9
c10
c11
c12
c13
c14
KYIPGTK+XL_S
KYIPGTK+XL_L
KTGQAAGFSYTDANK+XL_S
KTGQAAGFSYTDANK+XL_L
